# Improvement of Polylactide Properties through Cellulose Nanocrystals Embedded in Poly(Vinyl Alcohol) Electrospun Nanofibers

**DOI:** 10.3390/nano7050106

**Published:** 2017-05-11

**Authors:** Carol López de Dicastillo, Luan Garrido, Nancy Alvarado, Julio Romero, Juan Luis Palma, Maria Jose Galotto

**Affiliations:** 1 Food Packaging Laboratory (Laben-Chile), Department of Science and Food Technology, Faculty of Technology, Center for the Development of Nanoscience and Nanotechnology (CEDENNA), Universidad de Santiago de Chile (USACH), Obispo Umaña 050, 9170201 Santiago, Chile; luan.garrido.a@gmail.com (L.G.); maria.galotto@usach.cl (M.J.G.); 2 Laboratory of Membrane Separation Processes (LABPROSEM), Engineering Faculty, University of Santiago de Chile (USACH), Obispo Umaña 050, 9170201 Santiago, Chile; nalvarad8@gmail.com (N.A.); julio.romero@usach.cl (J.R.); 3 Engineering Faculty, Universidad Central de Chile, Santa Isabel 1186, 8330601 Santiago, Chile; juan.palma@ucentral.cl

**Keywords:** nanocomposite, nanocellulose, electrospinning, nanofiber, poly(lactic acid)

## Abstract

Electrospun nanofibers of poly (vinyl alcohol) (PV) were obtained to improve dispersion of cellulose nanocrystals (CNC) within hydrophobic biopolymeric matrices, such as poly(lactic acid) (PLA). Electrospun nanofibers (PV/CNC)*_n_* were successfully obtained with a final concentration of 23% (*w*/*w*) of CNC. Morphological, structural and thermal properties of developed CNC and electrospun nanofibers were characterized. X-ray diffraction and thermal analysis revealed that the crystallinity of PV was reduced by the electrospinning process, and the incorporation of CNC increased the thermal stability of biodegradable nanofibers. Interactions between CNC and PV polymer also enhanced the thermal stability of CNC and improved the dispersion of CNC within the PLA matrix. PLA materials with CNC lyophilized were also casted in order to compare the properties with materials based on CNC containing nanofibers. Nanofibers and CNC were incorporated into PLA at three concentrations: 0.5%, 1% and 3% (CNC respect to polymer weight) and nanocomposites were fully characterized. Overall, nanofibers containing CNC positively modified the physical properties of PLA materials, such as the crystallinity degree of PLA which was greatly enhanced. Specifically, materials with 1% nanofiber 1PLA(PV/CNC)*_n_* presented highest improvements related to mechanical and barrier properties; elongation at break was enhanced almost four times and the permeation of oxygen was reduced by approximately 30%.

## 1. Introduction

Facing the need to reduce the negative effects on the environment caused by the accumulation of conventional petroleum-based polymer waste, the efforts of material researchers have focused on the development of new materials based on biopolymers; these are degraded under natural conditions by microorganisms without leaving toxic or harmful waste in the environment. These biodegradable materials present a number of excellent and promising properties in several applications, including in packaging, and the automotive and biomedical sectors. Some of their properties need to be improved, such as excessive brittleness and insufficient barrier properties; however, the development of nanocomposites has been considered a promising solution to these disadvantages. A nanocomposite is a multiphase composite where at least one of the phases is present in the nanoscale dimension.

Over the last years, nanocellulose and particularly cellulose nanocrystals (CNCs) have attracted much interest for the production of fully-renewable and biodegradable nanocomposites. CNCs are natural nanofillers obtained from cellulose, a fibrous, hard, and water-insoluble substance that plays an essential role in maintaining the structure of plant cell walls. The multiple connections between cellulose chains through hydrogen bonding constitute cellulosic fibrils which have highly ordered (crystalline) and unordered (amorphous) regions. Amorphous regions can be selectively hydrolyzed through acid hydrolysis, to obtain nanosized crystalline regions called cellulose nanocrystals [1]. Many studies have focused on the isolation and characterization of CNCs from various sources of cellulose [2]. As compared to inorganic reinforcing fillers, CNCs have many additional advantages including the wide availability of sources, low-energy consumption, ease of recycling by combustion, high aspect ratio and good mechanical properties [3,4]. In addition, several studies in the last decade have associated the incorporation of CNCs with improvements in dynamic mechanical thermal properties, tensile strength, toughness and elongation at break [5,6,7,8]. Nevertheless, the use of CNCs as nano-reinforcement is a relatively new field in nanotechnology and, as a result, there are still many issues to be resolved and understood [4]. One of the main difficulties associated with the use of CNCs as reinforcing agents is their high hydrophilicity and strong hydrogen bond interactions, which makes them difficult to disperse in hydrophobic media including most widely researched thermoplastic biopolymers, such as poly(lactic acid) (PLA) and polyhydroxyalkanoates (PHAs). Thus, some strategies have been devised in order to improve the dispersion of CNCs into polymer matrices, such as grafting and chemical surface modification of CNCs, masterbatch in situ polymerization, the use of surfactants and partial silylation [9,10,11,12,13]. Nevertheless, most of these modifications are complicated processes and results have demonstrated that modified CNCs have less of a reinforcing effect. Thus, in this work, the alternative strategy proposed was the incorporation of CNCs into PLA by means of electrospinning with polyvinyl alcohol (PV). The principal aim was the development and the study of these nanofibers as an efficient strategy to successfully disperse CNCs into hydrophobic biopolymers and to obtain homogeneous nanocomposites in order to improve barrier and mechanical properties. PLA was the polymer selected because it is commercially available and produced on a large industrial scale. PV was selected because it is a water-soluble polymer that has two advantages. First of all, it is possible to be processed in water, and considering that PLA is extensively used in food packaging, it will not be a problem where other solvents might be an issue; and secondly, traditionally, cellulose nanocrystals are processed and highly dispersible in water, so the use of a water-soluble polymer will simplify the production. In addition, since PV polymer was directly dissolved into CNC obtaining solution, the incorporation of CNCs embedded into electrospun PV nanofibers can provide an effective way to eliminate the freeze-drying process of the CNC aqueous solution which is one of the longest and most energetically costly processes in the procedure for obtaining CNCs. Electrospinning is a simple and effective method of producing nanofibers whereby an electrical potential is applied between a droplet of a polymer solution held at the end of a capillary tube and grounded target. When the applied electric field overcomes the surface tension of the droplet, a charged jet of polymer solution is ejected and is controlled by the electric field. In recent years, the number of applications and research fields using this technique has increased significantly [14]. Martinez-Sanz et al. [15] have already incorporated bacterial cellulose nanocrystals into PLA through PLA electrospun fibers resulting in materials with higher values of tensile strength and elastic modulus, but lower elongation and barrier properties. The challenge of the present research is to improve the dispersion of CNC into PLA by using electrospun nanofibers of PV, a different polymer matrix with higher hydrophilic character. Additionally, PV is a non-toxic, biocompatible and biodegradable polymer that can be used in a wide range of applications in medical, cosmetic, food, pharmaceutical, and packaging industries. Moreover, some studies have also obtained successfully electrospun PV nanofibers [16,17].

Despite the lack of apparent compatibility between these two polymers, the results were interesting. The morphology, thermal, mechanical and barrier properties of PLA nanocomposites were studied and compared to PLA nanocomposites with freeze-dried CNCs. Moreover, the study of the effect of the electrospinning process and the incorporation of CNC in the PV properties were also investigated.

## 2. Experimental

### 2.1. Materials and Nanoreinforcements

#### 2.1.1. Polymers and Chemicals

Poly(lactic acid) (PLA), 2003D (specific gravity ¼ 1.24; MFR g/10 min (210 °C, 2.16 kg)) was purchased from Natureworks^®^ Co. (Minnetonka, MN, USA). Gohsenol type AH-17 polyvinyl alcohol (PV) (saponification degree 97–98.5% and viscosity 25–30 mPa·s) was obtained from The Nippon Synthetic Chemical Co. (Osaka, Japan). Cellulose fibers (CF) (powder 80–145 µm), and polyethylene glycol (PEG) were supplied by Sigma Aldrich. Chloroform and sulfuric acid 95–97% were supplied by Merck. Low-flow PES 170 dialysis membranes (35 µm thickness, 20.000 Da pores-size) were purchased from Nipro Medical Corporation (Santiago, Chile).

#### 2.1.2. Cellulose Nanocrystals Solution

Cellulose nanocrystals were prepared following the procedure of Bondenson et al. (2006) with some modifications [18]. Ten grams of cellulose fibers, CF, was mixed with 50 mL of deionized water and put in an ice bath and stirred while 50 mL of concentrated sulfuric acid were added dropwise until the solution achieved 9 M concentration. The suspension was then heated at 45 °C and stirred for 120 min, followed by the addition of water to stop the hydrolysis. The resulting mixture was centrifuged at 4000 rpm for 20 min, the clear supernatant containing acid residues and amorphous regions of the cellulose fiber was removed. Subsequently, successive washings were performed by adding 50 mL of distilled water and the tubes were shaken again and centrifuged at 4000 rpm for 12 min. This operation was repeated until the supernatant was a turbid suspension containing the CNC [18,19]. The suspension obtained was dialyzed until the washing water maintained at constant pH. A known volume of the previous CNC suspension was lyophilized to calculate the concentration of CNC and to obtain dry CNC to perform CNC nanocomposites used as control nanocomposites.

#### 2.1.3. Electrospun PV Nanofibers

CNC solution obtained from dialysis was concentrated through evaporation until a final concentration of 1.8% (*w*/*v*) owing to achieve a high incorporation degree of CNC into the electrospun PV nanofibers in order to incorporate the minimum concentration of PV in the final PLA nanocomposite. 1.2 g poly(vinyl alcohol) (PV) was added to 20 mL of CNC solution and stirred at 90 °C until polymer was dissolved. PV solution at the same concentration without CNC was also electrospun. Solutions were transferred to 5 mL plastic syringes and connected through a PTFE tube to a 18-gauge blunt stainless steel needle charged by a high voltage power supply with a range of 0–30 kV. The collector plate was fixed at a working distance of 10 cm below the needle tip and connected to the grounded counter electrode of the power supply. A voltage of approximately 10 kV and the flow rate of 0.25 mL/h were used. CNC containing PV nanofibers were named “(PV/CNC)*_n_*”, while neat PV nanofibers were named “(PV)*_n_*”.

### 2.2. PLA Nanocomposites Preparation

PLA based films were obtained by solution-extension-evaporation process (“casting”). Electrospun (PV/CNC)*_n_* were mixed with PLA solution in chloroform in order to obtain blends with a final concentration of 0.5%, 1% and 3% wt of CNC respect PLA weight and films were designed as “0.5PLA(PV/CNC)*_n_*, 1PLA(PV/CNC)*_n_* and 3PLA(PV/CNC)*_n_*”, respectively. Thus, it was necessary the incorporation of the electrospun nanofibers (PV/CNC)*_n_* at 2.2% for 0.5PLA(PV/CNC)*_n_*, 4.7% for 1PLA(PV/CNC)*_n_*, and 13% for 3PLA(PV/CNC)*_n_* (shown in Table 1). Control nanocomposite films with lyophilized CNC (“0.5PLACNC, 1PLACNC and 3PLACNC”) were also casted owing to study the effect of the encapsulation of CNC into PV electrospun nanofibers. A second series of control films including nanofibers with only PV, (PV)*_n_*, was also prepared in order to conclude the influence of PV on PLA properties. These control films were named 0.5PLA(PV)*_n_*, 1PLA(PV)*_n_* and 3PLA(PV)*_n_*. It is crucial to elucidate the effect of each component on the final PLA properties changes. PLA blank was also casted and named “PLA”. PEG was added at 5% *w*/*w* polymer for all formulations to facilitate the casting process. Table 1 shows the percentage of each component used to develop every film. Casting was done over a petri dish of 18 cm diameter and film drying was accomplished by using a stove at 60 °C during 1 h and left overnight at 40 °C. Subsequently, the films were removed from the plate and dried in a vacuum oven for 24 h. The thickness of every sample was individually measured using a digital micrometer with average values expressed in Table 1.

### 2.3. Characterization of Nanofibers, Cellulose Nanocrystals and PLA Nanocomposites

#### 2.3.1. Electron Microscopy (SEM and TEM)

The morphology of the electrospun nanofibers and the nanocomposites were studied using a scanning electron microscope (SEM) JSM-5410 Jeol (Tokyo, Japan) with accelerating voltage at 10 kV. Films were fracturated using Tensile Tester because it was not possible to obtain the samples through cryo-fracture. Then, samples were coated with gold palladium using a Sputtering System Hummer 6.2., and SEM micrographs of the surface and the cross-section of the materials were taken. Nanofibers were also analyzed through transmission electron microscopy (TEM). Nanofibers were placed on a cupper grid and examined under a Phillips Tecnai 12 Bio Twin TEM at 80 kV. Images were recorded using a CCD camera Olympus Megaview G2 at different magnifications.

#### 2.3.2. X-ray Diffraction (XRD)

Structures of CNC, nanofibers and PLA nanocomposites were evaluated with X-ray diffraction (XRD). XRD patterns were measured using a Siemens Diffractometer D5000 (Erlangen, Germany) with 30 mA and 40 kV using CuKa (λ = 1.54 Å) radiation at room temperature. All scans were performed in a 2θ range 2–12° at 0.02°/s.

#### 2.3.3. Thermal Properties

Thermogravimetric analyses (TGA) were carried out using a Mettler Toledo Gas Controller GC20 Stare System TGA/DCS (Schwerzenbach, Switzerland)*.* Samples (ca. 9 mg) were heated from 20 to 600 °C at 10 °C min^−1^ under nitrogen atmosphere (flow rate 50 mL·min^−1^).

Differential Scanning Calorimetry (DSC) analyses were also performed with a Mettler Toledo DSC-822e calorimeter (Schwerzenbach, Switzerland). Thermograms were obtained from −20° to 220°, cooling to −20 °C, and a second heating process to 220 °C with 10 °C min^−1^ heating rate. Sample weight was about 8–10 mg. The degree of crystallinity (*X_c_*) of the PLA materials was deduced using the equation :

*X_c_* = % crystallinity of PLA = 100 × [(Δ*H_m_* − Δ*H_cc_*)/Δ*H*^0^*_m_*]

where Δ*H_m_* is the specific melting enthalpy of the sample (J g^−1^); Δ*H_cc_* is the specific cold crystallization enthalpy of the sample (J g^−1^) and Δ*H*^0^*_m_* is the specific melting enthalpy of a wholly crystalline PLA (93.1 J g^−1^) [20].

#### 2.3.4. Testing 

Tensile testing of each material was measured using a Zwick Roell model BDOFB 0.5 TH Tensile Tester (Ulm, Germany), according to ASTM D-882. Strips (10 cm × 2.5 cm) of films were cut using a die cutter and kept at 25 °C and 50% RH for 48 h before the test. Analyses were carried out with a 1 kN load cell. The initial grip separation was 10 cm and the crosshead speed used was 50 mm·min^−1^. Results are the average of 8 specimens for each film.

#### 2.3.5. Oxygen Permeability

The oxygen permeation rates of the materials were determined at 0%, 35%, 50% and 75% relative humidity (RH), and 23 °C using an Oxtran model 2/21 ML Mocon (Lippke, Neuwied, Germany). Films were previously purged with nitrogen for a minimum of 16 h in the RH desired, prior to exposure to an oxygen flow of 10 mL/min. Permeation values were determined every 45 min until constant.

### 2.4. Statistical Analysis

A randomized experimental design was considered for the experiments. Data analysis was carried out using Statgraphics Plus 5.1 (StatPoint Inc., Herndon, VA, USA). This software was used to implement variance analysis and Fisher’s LSD test. Differences were considered significant at *p* < 0.05.

## 3. Results and Discussion

### 3.1. Morphological Results of Nanostructures and Nanocomposites

**Figure 1:**
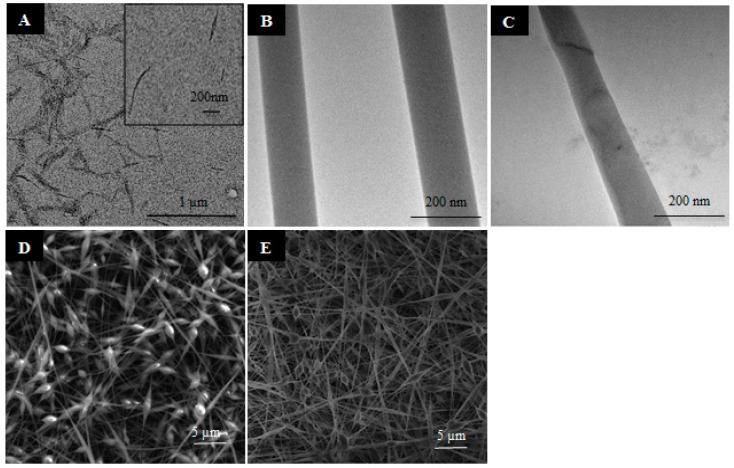
Morphology of nanofibers and cellulose nanocrystal (CNC) analyzed with transmission electron microscopy (TEM): (**A**) CNC; (**B**) (PV)*_n_*; (**C**) (PV/CNC)*_n_*; and SEM microscopy: (**D**) (PV)*_n_*; (**E**) (PV/CNC)*_n_*.

(PV/CNC)*_n_* were successfully obtained with a final relation of 23/77 (*w*/*w*) CNC/PV. TEM and SEM microscopies were useful tools to observe the morphology of CNCs and the electrospun nanofibers (PV)*_n_* and (PV/CNC)*_n_* (Figure 1A–E). As Figure 1A shows, CNCs produced by hydrolysis presented diameters of about 20 nm and lengths between 400 and 700 nm, which resulted in a high “aspect ratio”, a property which is essential to obtain a good reinforcing agent [20]. The morphology of electrospun nanofibers is shown in Figure 1B–E. SEM images (Figure 1D,E) revealed fibers presented diameters between 80 and 120 nm, and the distribution was relatively uniform, even the presence of some beads was difficult to avoid due to the low concentration of PV used. Cellulose nanocrystals did not affect the size of the fibers, and the homogeneity of the morphology was improved presenting fewer beads. TEM analysis also gave evidence that CNCs were completely embedded in the PV matrix, as shown in Figure 1C. As already observed in other previous studies, CNCs could have been aligned along the fiber axis under the electrical field produced during the electrospinning process [21,22].

**Figure 2:**
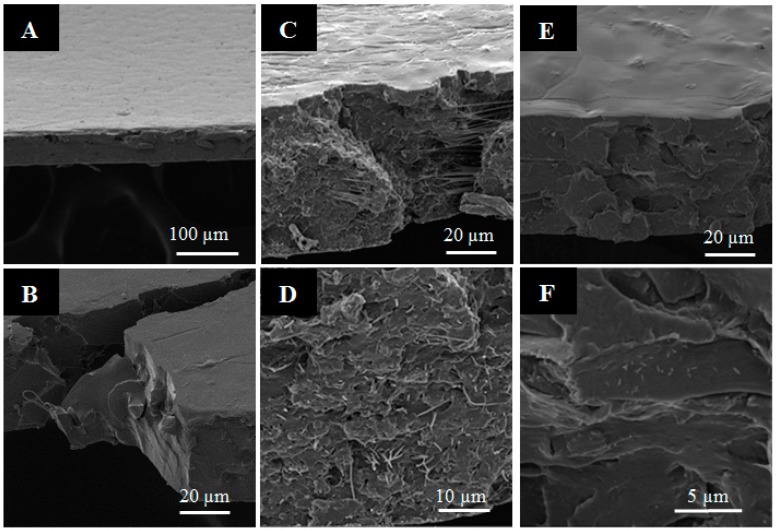
SEM images of poly(lactic acid) (PLA) nanocomposites: (**A**,**B**) PLA neat; (**C**,**D**) 1PLA(PV/CNC)*_n_*; and (**E**,**F**) 1PLACNC.

The morphology and the dispersion of the nanofibers and CNC in the PLA nanocomposites were studied through SEM microscopy. Electrospun nanofibers were incorporated into PLA and properties of these nanocomposites were compared to the properties of casted nanocomposites based on lyophilized CNCs. Micrographs of material surfaces and cross-sections of PLA control and nanocomposites with 1% nanofibers and CNC are presented, as an example, in Figure 2. The PLA control sample exhibited a smooth surface and apparently a compact and homogeneous structure (Figure 2A,B). Nanocomposites with the direct incorporation of CNCs presented a smooth surface similar to the control sample, although it was possible to observe certain agglomerations when CNC concentration increased (see Figure 2E,F). Meanwhile, the films reinforced with nanofibers loaded with CNC (PV/CNC)*_n_* at lowest concentration (film 0.5PLA(PV/CNC)*_n_*) had a smooth surface, but, at higher concentrations (films 1PLA(PV/CNC)*_n_* and 3PLA(PV/CNC)*_n_*), it was possible to distinguish two different surfaces due to the process used: ‘casting’. The surface, which was in contact with the glass during casting, presented also a smooth surface while the other side presented some roughness (shown Figure 2C), probably due to some nanofiber agglomeration. Nevertheless, the cross-section images showed the homogeneous dispersion of nanofibers containing CNCs through film thickness (see Figure 2D). Similarly, nanofibers at higher concentration were uniformly dispersed into the PLA matrix.

### 3.2. X-ray Analysis Results

**Figure 3:**
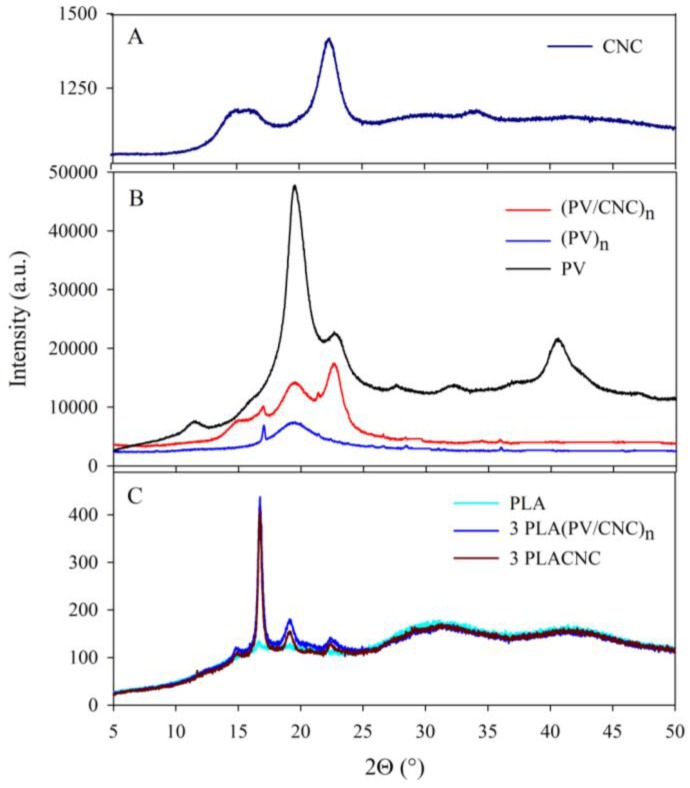
X-ray diffraction patterns of: (**A**) cellulose nanocrystals (CNC); (**B**) PV electrospun nanofibers and PV polymer; and (**C**) PLA nanocomposites.

Evaluations with X-ray diffraction (XRD) of cellulose nanocrystals and nanofibers (CNC and (PV/CNC)*_n_*) and nanocomposites at the highest concentration of these nanocomponents, as an example, are plotted in Figure 3. The diffraction pattern of CNCs exhibited a sharp peak at 2θ = 22.34°, corresponding to the crystallographic plane 002, and the cellulose shoulder at 2θ = 15.5°, which is normally assigned to the cellulose I structure [23,24]. PV polymer control (not processed through electrospinning) was also analyzed, with the aim of studying the effect of the electrospinning process in the crystallinity of this polymer. The PV diffraction pattern presented characteristic peaks at 2θ = 11.5°, 19.5°, 22.6°, 32.1°, and 40.5° which were attributed to the semi-crystalline nature of the polymer [25,26,27,28]. As Figure 3B shows, XRD of electrospun PV, (PV)*_n_*, presented only the main crystallographic peak at 2θ = 19.5°, confirming that the electrospinning process changed PV crystallinity. Certainly, the crystallization process during electrospinning implied the generation of different spherulite sizes, although crystallinity degree remained the same (as shown later in DSC results). On the other hand, the (PV/CNC)*_n_* diffraction pattern exhibited two PV characteristic peaks (2θ = 19.5° and 22.4°), probably because the presence of CNC favored the polymer crystallization during the electrospinning process. In addition, a new peak at 2θ = 15.5° appeared, which was attributed to the presence of CNC.

The XRD analyses of the developed materials were performed to obtain information about material crystallinity (Figure 3C). The PLA control exhibited a small peak at 2θ = 16.7°. Other studies have shown that PLA can present several characteristic bands to the semicrystalline nature of the polymer [29], but the incorporation of PEG, as a plasticizer, decreased the crystallinity. Nevertheless, as Figure 3C shows, the incorporation of CNCs and nanofibers (PV/CNC)*_n_* enhanced the degree of crystallinity of PLA, with new diffraction peaks appearing at 2θ = 14.9°, 19.1°, and 22.5°, which agreed with data obtained by Pagés et al. [29]. With regard to the polymorphism of PLA, according to Pan et al., during PLA crystallization in solution, polymer chains have sufficient mobility and, therefore, tend to reach a state of equilibrium, which favors the formation of thermodynamically stable α crystals, instead of the kinetically controlled α′ (δ) crystals. This fact was confirmed in Figure 3C in which the characteristic diffraction peak of the (δ) crystals α′ at 2θ ≈ 24.5° was not displayed [30].

### 3.3. Thermal Properties of CNC, Nanofibers and Developed Nanocomposites

**Figure 4:**
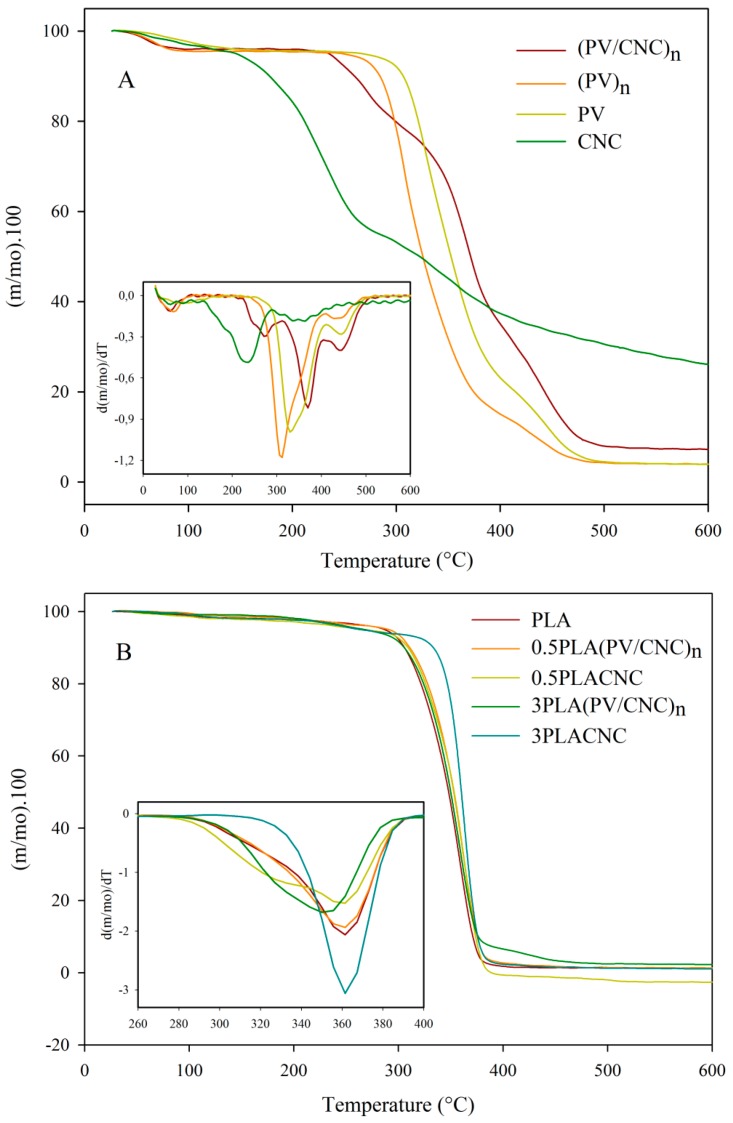
(**A**) DTGA (derivative thermogravimetric analysis) curves of individual components; (**B**) DTGA of PLA nanocomposites.

Figure 4 shows the TGA curves of mass loss and their derivative values with temperature, and Table 2 presents the temperature values at maximum degradation for all nanocomposites. As was expected, and seen in previous works, CNCs presented an earlier degradation of cellulose chains due to the presence of residual sulphate groups remaining from the acid hydrolysis that generated degradation through catalysis by dehydration [31,32]. As Figure 4A shows, the first maximum degradation of CNCs occurred approximately at 231 °C corresponding to cellulose depolymerization and decomposition of glycosyl units, followed by the oxidation and decomposition of carbonized residues [32]. The TGA curve of PV control was included to observe the effect of the electrospinning process on thermal properties. PV presented two distinguished decomposition processes, with maximum degradation temperatures at 337 °C and 437 °C. The first degradation process was related to the separation of side groups which form water, acetic acid and acetaldehyde byproducts, while the second was associated to a decomposition of the main polymer chains of PV [33]. Normally, the main degradation is associated with the crystalline polymeric section, and the continuing shoulder is related to degradation in the molten state [34]. Meanwhile, PV nanofibers, (PV)*_n_*, presented an earlier degradation with a maximum at approximately 305 °C attributed to the electrospinning process causing a change in the polymer nanoscale structure, which meant that it had more surface and, hence, the heat penetrated faster. On the other hand, nanofibers containing CNC, (PV/CNC)*_n_*, presented better thermal stability. As Figure 4A shows, there was an increase of approximately 20 degrees of *T_onset_* and the temperature of maximum degradation of PV nanofibers containing CNC due to the formation of hydrogen bonds between PV side chains and CNCs [35]. In addition, a peak at 270 °C, which was preceded by a shoulder, corresponded to the degradation of CNCs. Thermal stability of CNCs was also improved due to these interactions.

Regarding the nanocomposites, they all presented one main degradation process which indicated good compatibility between the constituents of the films. In general, the addition of nanofibers (PV/CNC)*_n_* to the PLA matrix slightly decreased the thermal stability of nanocomposites, probably due to the earlier degradation of the PV polymer matrix. As concentration of (PV/CNC)*_n_* increased, temperature of maximum degradation shifted to lower temperatures, although these decreases were not significantly different. On the other hand, nanocomposites with CNC did not present significant changes on the maximum degradation temperature but, as Figure 4B shows, the incorporation of CNC changed the thermal behavior of the PLA matrix. Interestingly, in the case of nanocomposite 3PLACNC, the peak of maximum degradation of PLA turned sharper and the shoulder of degradation seen on other nanocomposites and the PLA control disappeared (as shown in Figure 4B).

**Figure 5:**
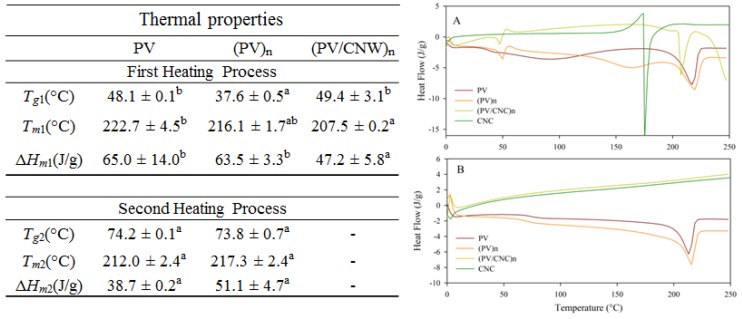
Left: Thermal parameters of PV polymer and PV electrospun nanofibers during first and second heating process Lower case letters a–d indicate significant differences in a thermal parameter among the samples. ‘a’ corresponds to the smaller values and ‘b’ the higher ones. Right: Differential Scanning Calorimetry (DSC) thermograms during (**A**) first and (**B**) second heating processes of poly (vinyl alcohol) (PV), electrospun nanofibers and CNC.

In addition, DSC thermograms were performed to deduce, firstly, the effect of the incorporation of CNC and the electrospinning process on PV crystallinity, and, secondly, the effect of the incorporation of cellulose nanocrystals and electrospun nanofibers on PLA thermal properties. In the case of electrospun nanofibers, both the first and second heating processes were analyzed. As Figure 5A shows, during the first heating process, the CNC thermogram presented an endothermic peak at 175 °C attributed probably to the melting process of the CNC crystals. Nevertheless, during the first heating process, some degradation of cellulose occurred, confirmed by TGA analysis (Figure 4A) and because any enthalpy was observed during cooling and second heating processes (Figure 5B).

The electrospinning process also influenced PV thermal properties since the first heating process showed a decrease on glass transition temperature, *T_g_* value, while crystallinity was not significantly affected. Surely, the decrease on *T_g_* value was due to the presence of residual water, confirmed by the mass loss on TGA analysis, that resulted in a plasticizing effect. Therefore, after removing the thermal history of the materials, *T_g_* values of the PV control and electrospun nanofibers were not significantly different. Nevertheless, melting enthalpies, which were initially similar, during second heating process, melting enthalpy of (PV)*_n_* nanofibers was greatly higher than PV control, probably due to the lower degradation suffered during the first heating process.

CNC incorporation into PV nanofibers also had an interesting effect. Electrospun (PV/CNC)*_n_* presented an enhancement of *T_g_* value, indicating that the mobility of the chains of the polymer was reduced by the presence of the CNCs due to the interactions between PV and the CNCs. In addition, CNC incorporation affected the crystallinity conformation because any enthalpic process was observed during the cooling and second heating processes, suggesting the first heating process completely degraded the crystal structure. As Figure 5A shows, after exothermic melting enthalpy, (PV/CNC)*_n_* started suffering a degradation process that was confirmed by running a DSC of this sample until 300 °C.

Table 2 summarizes all thermal properties obtained for PLA nanocomposites during the second heating process where enthalpies (Δ*H_cc_* and Δ*H_m_*) were corrected for PLA content. Nanocomposites showed a slight decrease on glass transition temperatures, cold crystallization and melting temperatures associated with the plasticizing effect of (PV/CNC)*_n_* nanofibers [13]. As SEM images showed, as in Figure 2B, electrospun nanofibers (PV/CNC)*_n_* were well dispersed and probably intermingled between polymer chains enhancing their mobility. However, when nanofiller concentration was 3%, this trend changed and the rigidity of materials increased. Nanofibers (PV/CNC)*_n_* facilitated the crystallization process of PLA biopolymer. This fact was already observed with the XRD results. Other works have found that cellulose fibers induced crystal nucleation at the fiber surface, and this effect was called the transcrystallinity effect.

This result is very positive from a barrier perspective, since crystals are typically impermeable systems but, mechanically, could be negative because crystallization can promote additional rigidity and, hence, fragility for the biopolymer mechanical performance [5,36,37]. Roohani et al. have also reported that the interactions between the cellulosic surface and polymeric matrix can restrict the capability of the polymer chains to grow larger crystalline domains [20,38]. As Table 2 shows, when CNCs were ‘encapsulated’ this effect was certainly inhibited and crystallinity was enhanced.

### 3.4. Mechanical Properties

The mechanical properties of the films were characterized by Young’s modulus (YM), tensile strength (TS), and elongation at break (EB) values and the results are summarized in Table 3. YM, TS and EB values of PLA were in agreement with those reported in other works [13,15,39].

In general, the incorporation of electrospun nanofibers (PV/CNC)*_n_* increased YM, although statistical analysis revealed the results were not significantly different. As already observed on DSC analysis, the presence of (PV/CNC)*_n_* induced an extra polymer crystallization that could result in this enhancement of Young’s modulus because the material also increased their rigidity. Moreover, the presence of these nanofibers increased considerably the elongation at break values of these nanocomposites. Although this enhancement on EB values was due to the presence of PV electrospun fibers since composites with (PV)*_n_* also presented some increase, this effect was greatly higher in the nanocomposites with CNC-containing electrospun nanofibers. EB value of 1PLA(PV/CNC)*_n_* material was approximately three times higher than those values published in different literature studies [3,4,9,13,15,39]. This result is remarkable since several works have shown the brittle behavior of neat PLA and how the lack of interaction between CNCs and PLA induced a reduction of mechanical properties, mainly EB values [9,13,15]. In this work, the good compatibility between CNCs and PV, probably generating the occurrence of hydrogen bonding interactions between CNCs and PV, their subsequent good dispersion of these electrospun nanofibers and the interactions between nanofibers and PLA matrix, the ability of these nanofibers to organize and reorient and the mechanical strength of CNCs are the reasons of this great improvement on PLA performance. Martinez-Sanz et al. have also mentioned the fact that when strong interactions, such as hydrogen bonding, take place between the matrix (PV) and the nanofiller (CNCs), the stress concentration effect is prevented to a certain extent due to an effect referred to as reinforcing plasticizing phenomenon values [15]. Regarding nanocomposites with freeze-dried CNC, EB results were clearly decreased, probably, although the addition of reinforcing agents act as stress components, because the agglomeration of CNC resulted in break points of these materials.

Mechanical properties of control films based on PV nanofibers without CNC, (PV)*_n_*, were also performed in order to study the influence of PV on the modification of these parameters. As Table 3 shows, films with (PV)*_n_* presented similar effects than nanofibers with embedded CNCs, excepting for elongation at break values, where the presence of CNCs considerably increased this parameter. Arrieta et al. already observed a similar effect when the modification of CNCs improved their dispersion into PLA-PHB materials and generated better interaction between PLA and PHB [40].

Ultimately, the TS value showed a slight decrease with respect to PLA. The reduction of TS have occurred in previous works and the values are within the range reported by other authors, which are between 30 and 60 MPa [9,13,15,19,20]. Nevertheless, this effect was reduced when CNCs were embedded into PV nanofibers. This behavior was possibly related with the homogeneous dispersion of the nanofibers in the polymeric matrix which resulted in an increase of ductility. On the other hand, YM, and EB parameters of CNC-containing nanocomposites were oppositely affected, possibly due to the lack of dispersion and CNC agglomeration, mechanical properties of nanocomposites were not improved. As was shown in other works, nanocomposites with CNCs presented a reduction in Young’s modulus caused by a stronger softening effect of the CNCs, accompanied by a decrease of TS and EB [13,15,20].

### 3.5. Oxygen Permeation Results

**Figure 6:**
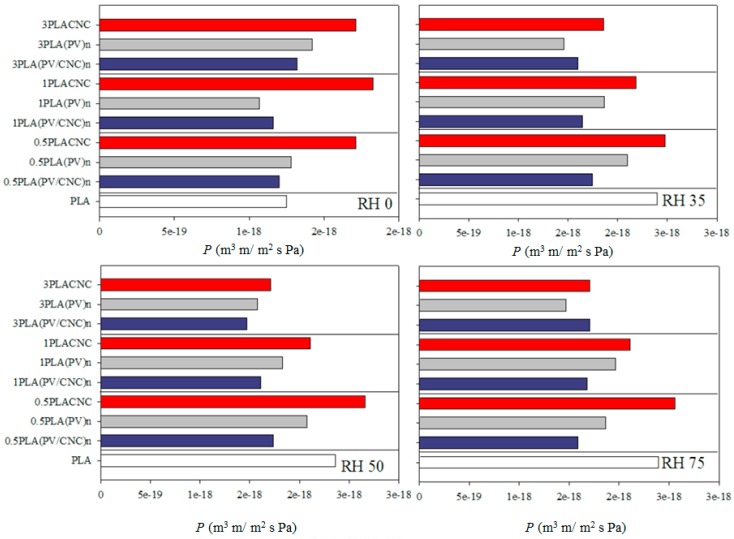
Oxygen permeation for PLA-based materials at different relative humidities.

Figure 6 gathers the oxygen permeability values of PLA materials measured at different RH. Permeability results presented values between 8 × 10^−19^ and 3 × 10^−18^ m^3^ m/s m^2^ Pa, which are in agreement with those reported in the literature for PLA [15,20,41]. The reason for the differences could be related to the different obtaining processes and origins of the samples.

In general, transport properties are known to be strongly influenced by tortuous path altering factors, including shape and aspect ratio of the filler, degree of dispersion, filler loading, orientation, adhesion to the matrix, moisture activity, filler-induced crystallinity, polymer chain immobilization, and filler induced solvent retention [4,8,12]. Thus, when CNC or electrospun nanofibers had better dispersion and adhesion to the matrix, a higher barrier property was shown.

As Figure 6 shows, materials with CNC incorporated through PV electrospun nanofibers (blue bars) presented better performance than freeze dried CNC-based nanocomposites (red bars). Specifically, 1PLA(PV/CNC)*_n_* was the nanocomposite that exhibited the highest oxygen barrier, mainly at high RH. Indeed, this material showed a reduction in the oxygen permeation approximately of 30% at 35%, 50% and 75% RH. In general, permeation values of PLA nanocomposites with (PV/CNC)*_n_* were very similar to results obtained by Martinez-Sanz et col. with oxygen permeability values ranging from 1.5 × 10^−18^ to 2.2 × 10^−18^ m^3^ m/s m^2^ Pa [15]. Permeation results were in accordance with thermal data discussed before related to the crystallinity rise due to nanofiller-induced nucleation. Nevertheless, nanocomposites with highest concentration of CNC embedded into PV nanofibers did not present the expected improvement. Probably, the presence of residual solvent and the nanofiber clumping observed when nanofibers were incorporated at high concentration were the main reasons of these results. A second feasible explanation is the specific morphology of the solvent-cast polymeric films that hindered the expected improvement.

The effect of the incorporation of the PV polymeric matrix in the formulation of these nanocomposites is worthy of highlighting. As shown in Figure 6, control materials with (PV)*_n_* (grey bars) also presented a decrease on oxygen permeability values. The presence of PV nanofibers contributed to the improvement of barrier properties but, mainly in the best performance material, the presence of CNCs also played an important role. Probably, the strong interactions between CNCs and PV based on hydrogen bonding (already shown in mechanical properties) decreased the effect of humidity on PV polymer due to the reduction of available PV hydroxyl groups to interact with water.

On the other hand, CNCs-containing nanocomposites presented higher values of oxygen permeation than when compared to the PLA control. Contrary to other works, nanocomposites with freeze-dried CNCs did not present improvement on barrier properties [13,15]. Although thermal results have shown enhancement of crystallinity, permeation was not reduced. This fact was due to the lack of dispersion of cellulose nanocrystals and, possibly, the appearance of gas passage points due to the CNC agglomeration.

Another interesting fact to highlight was that oxygen permeation was not affected by water activity. In some cases, gas permeability was lower when relative humidity was very high, and no significant differences were observed between RH. This effect has already been reported [15,41]. The explanation is a combination of increased diffusion and decreased solubility. Although the PLA oxygen diffusion coefficient increases exponentially with water activity due to plasticization of the amorphous phase by water molecules, the solubility coefficient decreases linearly with water activity as a consequence of the occupancy of the free volume by water molecules [41,42].

## 4. Conclusions

The main goal of this study was to improve the dispersion of cellulose nanowhiskers (CNCs) within PLA matrices in order to improve its physical properties. The strategy used was the incorporation of CNCs into electrospun nanofibers of polyvinyl alcohol, (PV/CNC)*_n_*. Results were not so striking due to the presence of PEG as the plasticizer and, principally, the effect of the obtaining process. On the other hand, the characterization of PLA nanocomposites revealed that the incorporation of electrospun nanofibers with and without CNC considerably affected the principal properties. Electronic microscopy revealed it was possible to obtain a high dispersion of CNCs when they were incorporated into PLA matrix by means of PV electrospun nanofibers. There were some improvements in the properties of the nanocomposite materials compared to pure PLA. Results indicated that the incorporation of (PV/CNC)*_n_* modified the structural properties of PLA, and the performance of CNCs on the film for reinforcement is better through electrospun nanofibers. It was clearly evidenced that the incorporation of PV also influenced PLA properties. The films with electrospun nanofibers containing CNCs presented an improvement on the mechanical behavior, resulting in a more flexible and tough film. The ductility of the nanocomposite with electrospun nanofibers containing CNCs was clearly improved. Although it was not observed for all nanocomposites, barrier properties were significantly enhanced with the incorporation of CNCs through electrospun PV nanofibers at 1% when compared to CNCs-containing nanocomposite films. Undoubtedly, the material 1PLA(PV/CNC)*_n_* presented the best performance on mechanical and oxygen barrier properties. The performance of these novel nanocomposites was a sum of the effect of the incorporation of PV nanofibers and the presence of CNCs.

## Figures and Tables

**Table 1:** Composition (%) of developed films.

Film Samples	PLA	(PV/CNC)*_n_*	(PV)*_n_*	CNC	Thickness (µm)
PLA	100	-	-	-	66 ± 3 ^a^
0.5PLA(PV/CNC)*_n_*	97.8	2.2	-	-	71 ± 3 ^ab^
0.5PLA(PV)*_n_*	98.3	-	1.7	-	68 ± 4 ^ab^
0.5PLACNC	99.5	-	-	0.5	68 ± 6 ^ab^
1PLA(PV/CNC)*_n_*	95.7	4.7	-	-	67 ± 2 ^ab^
1PLA(PV)*_n_*	96.7	-	3.3	-	68 ± 4 ^ab^
1PLACNC	99.0	-	-	1.0	68 ± 7 ^ab^
3PLA(PV/CNC)*_n_*	87.0	13.0	-	-	69 ± 6 ^ab^
3PLA(PV)*_n_*	90.0	-	10.0	-	68 ± 5 ^ab^
3PLACNC	97.0	-	-	3.0	75 ± 7 ^b^

Lower case letters a–d indicate significant differences in thickness among the samples. ^a^ corresponds to the smaller values and ^b^ the higher ones.

**Table 2:** Thermal properties of PLA-based developed films.

Films	*T_deg_*_._	*T_g_* (°C)	*T_cc_* (°C)	Δ*H_cc_* (J/g)	*T_m_* (°C)	Δ*H_m_*(J/g)	*X*_c_′ (%)
PLA	365.6 ± 2.1 ^a^	39.0 ± 1.4 ^bc^	90.6 ± 0.2 ^bc^	25.7 ± 0.2 ^c^	148.2 ± 0.8 ^bc^	−30.3 ± 0.4 ^b^	3.2 ± 1.4 ^a^
0.5PLA(PV/CNC)*_n_*	362.1 ± 0.3 ^a^	36.6 ± 0.4 ^ab^	87.9 ± 0.2 ^ab^	22.2 ± 0.1 ^a^	146.3 ± 0.3 ^a^	−28.9 ± 1.1 ^b^	6.9 ± 0.8 ^a^
0.5PLACNC	363.1 ± 1.8 ^a^	35.4 ± 0.4 ^a^	85.4 ± 0.5 ^a^	24.6 ± 1.1 ^abc^	146.1 ± 0.6 ^a^	−28.9 ± 1.2 ^b^	4.7 ± 0.1 ^b^
1PLA(PV/CNC)*_n_*	361.5 ± 1.1 ^a^	37.8 ± 1.9 ^bc^	86.9 ± 3.3 ^ab^	22.6 ± 0.4 ^ab^	146.5 ± 1.3 ^a^	−29.0 ± 0.4 ^b^	6.8 ± 0.8 ^a^
1PLACNC	365.2 ± 0.1 ^a^	38.4 ± 3.7 ^bc^	95.2 ± 1.6 ^d^	25.4 ± 2.6 ^bc^	148.6 ± 0.2 ^bc^	−26.9 ± 0.7 ^c^	3.6 ± 0.7 ^b^
3PLA(PV/CNC)*_n_*	358.5 ± 0.5 ^a^	40.3 ± 0.8 ^c^	90.8 ± 2.5 ^bc^	24.0 ± 1.1 ^abc^	147.8 ± 0.7 ^ab^	−30.0 ± 0.1 ^b^	6.4 ± 1.2 ^a^
3PLACNC	365.4 ± 0.5 ^a^	38.5 ± 0.8 ^bc^	92.5 ± 0.8 ^cd^	29.6 ± 0.4 ^d^	149.7 ± 0.4 ^c^	−32.6 ± 0.2 ^a^	4.6 ± 1.1 ^b^

Lower case letters a–d indicate significant differences in a thermal parameter among the materials. ^a^ corresponds to the smaller values and ^b^ the higher ones.

**Table 3:** Mechanical properties of developed PLA-based films.

Material	Young’s Modulus	Tensile Strength	Elongation at Break
	(GPa)	(MPa)	(%)
PLA	1.61 ± 0.16 ^b^	47.9 ± 4.6 ^de^	3.4 ± 0.4 ^ab^
0.5PLA(PV/CNC)*_n_*	1.66 ± 0.16 ^b^	52.0 ± 2.2 ^f^	4.3 ± 0.8 ^ab^
0.5PLA(PV)*_n_*	1.54 ± 0.16 ^b^	51.2 ± 2.7 ^f^	5.9 ± 1.6 ^bc^
0.5PLACNC	1.61 ± 0.13 ^b^	40.3 ± 2.2 ^b^	2.8 ± 0.4 ^ab^
1PLA(PV/CNC)*_n_*	1.82 ± 0.16 ^b^	45.3 ± 2.3 ^cd^	12.3 ± 4.6 ^d^
1PLA(PV)*_n_*	1.88 ± 0.13 ^b^	44.7 ± 2.5 ^cd^	5.8 ± 1.1 ^bc^
1PLACNC	1.12 ± 0.25 ^a^	41.9 ± 4.2 ^bc^	2.9 ± 0.6 ^ab^
3PLA(PV/CNC)*_n_*	1.72 ± 0.14 ^b^	40.2 ± 1.2 ^b^	8.3 ± 2.5 ^c^
3PLA(PV)*_n_*	1.77 ± 0.11 ^b^	37.2 ± 2.2 ^b^	5.6 ± 0.9 ^abc^
3PLACNC	1.24 ± 0.22 ^a^	31.2 ± 3.3 ^a^	2.6 ± 0.3 ^a^

Lower case letters a–d indicate significant differences in a mechanical parameter among the materials. ^a^ corresponds to the smaller values and ^d^ the higher ones.

## Abbreviations

PLA	Poly(lactic acid)
PV	Poly(vinyl alcohol)
CNC	Cellulose nanocrystals
PEG	Polyethylene glycol
(PV)*_n_*	Poly(vinyl alcohol) electrospun nanofibers
(PV/CNC)*_n_*	PV electrospun nanofibers containing CNC
PLA(PV)*_n_*	Nanocomposites of PLA containing nanofibers (PV)*_n_*
PLA(PV/CNC)*_n_*	Nanocomposites of PLA containing nanofibers (PV/CNC)*_n_*
PLACNC	Nanocomposites of PLA containing CNC
